# PCB Drill Path Optimization by Combinatorial Cuckoo Search Algorithm

**DOI:** 10.1155/2014/264518

**Published:** 2014-02-23

**Authors:** Wei Chen Esmonde Lim, G. Kanagaraj, S. G. Ponnambalam

**Affiliations:** ^1^Advanced Engineering Platform and School of Engineering, Monash University Sunway Campus, 46150 Bandar Sunway, Selangor, Malaysia; ^2^Department of Mechanical Engineering, Thiagarajar College of Engineering, Madurai 625015, India

## Abstract

Optimization of drill path can lead to significant reduction in machining time which directly improves productivity of manufacturing systems. In a batch production of a large number of items to be drilled such as printed circuit boards (PCB), the travel time of the drilling device is a significant portion of the overall manufacturing process. To increase PCB manufacturing productivity and to reduce production costs, a good option is to minimize the drill path route using an optimization algorithm. This paper reports a combinatorial cuckoo search algorithm for solving drill path optimization problem. The performance of the proposed algorithm is tested and verified with three case studies from the literature. The computational experience conducted in this research indicates that the proposed algorithm is capable of efficiently finding the optimal path for PCB holes drilling process.

## 1. Introduction

In the manufacturing industry, hole-drilling operation is almost always unavoidable. In particular in electronic manufacturing, drilling holes on the printed circuit board (PCB) is one of the most crucial processes. With the escalating growth in demand for computers and electronic gadgets, PCB assembly has undoubtedly become a competitive market. The increasing variety of products in need of PCB has made it inevitable for circuit board manufacturing industry to automate the hole-drilling operations. Many industries have adopted the computer numerical control system in automating the hole-drilling operations because of its control flexibility. In all machining processes, time is spent on both positioning the cutting tool and carrying out the machining operation and the hole-drilling operation is of no exception.

Due to the point-to-point tool movement in hole making and requirement of different tools for making each hole, a considerable amount of the processing time is spent on switching tools and moving the table from one location to another. The survey by Merchant [1] reported that tool movement and switching time take 70% of the total time in a manufacturing process, on average. Therefore, optimization of hole making operations can lead to significant reduction in machining time which directly improves productivity of manufacturing systems [2]. A crucial issue in manufacturing is cost effectiveness. Production costs must be minimized if the product is to compete in the marketplace. Maximizing the production of products that meet customer requirements is the prime objective of a manufacturing enterprise. Therefore, any strategy that can be adopted to minimize the production time will have much impact on achieving the firm's objectives [3].

Due to the significant amount of time required for moving the drill bit from one point to another, holes drill routing optimization problem attracts a great interest among the academicians, researchers, and engineers to solve it. This concern for calculation of the minimum tool path length between holes (the drilling device has to be steered to the location of each hole exactly once) is similar to a very well-known problem from the operational research field called the (symmetric, single objective, and Euclidean) traveling salesman problem (TSP). The problem of finding the best way the points to be drilled are traversed can be modeled as TSP in order to cut down the production cost. In this case, the holes to be drilled are the cities, and the cost of travel is the time it takes to move the drill head from one hole to the next. The routing of production through a manufacturing facility has often been identified as a TSP [4]. Tong et al. [5] indicated that, during leather machining, when punching holes in the surface of the leather, the machining sequence optimization of the holes is similar to TSP.

Despite the importance of drilling path optimization, few researchers worked on this problem in the literature. First, Kolahan and Liang [6] have formulated the problem as TSP and worked on applying tabu search algorithm to solve the problem, and then they extended their research to a more complex case  [7]. Consequently, several researchers have demonstrated the applicability of TSP techniques to the drilling path optimization problem over the past few years. The drilling path optimization has been solved using genetic algorithm [8], particle swarm optimization [3], global convergence particle swarm optimization [9], and ant colony system [10, 11]. Ghaiebi and Solimanpur [2] have used ant colony algorithm. Krishnaiyer and Cheraghi [12] used the same algorithm and they proposed a web base system. Adam et al. [13] have used particle swarm algorithm, and then Zhu and Zhang [9] have extended the research on drill path optimization problem and applied global convergence particle swarm optimization algorithm to obtain the global optimization solution.

**Pseudocode 1:**
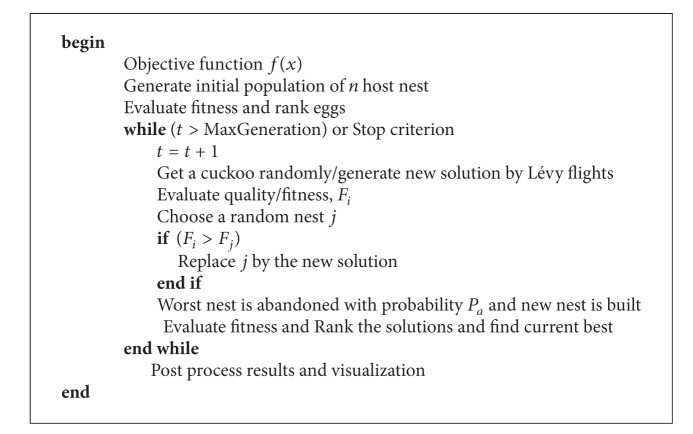
Pseudocode of the cuckoo search algorithm.

**Pseudocode 2:**
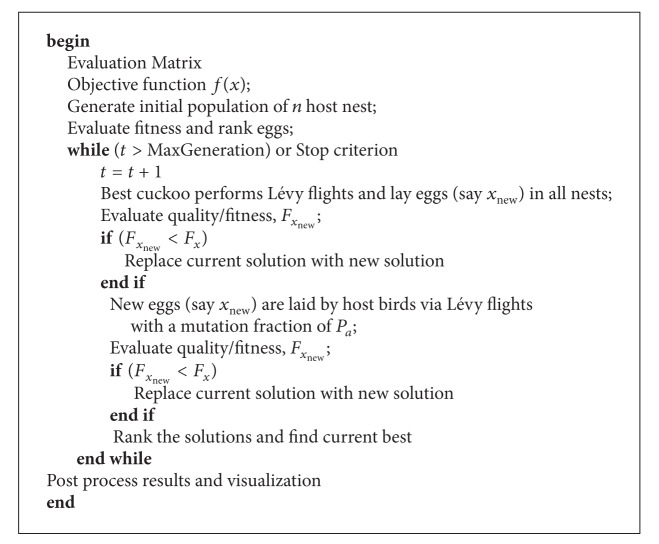
Pseudocode of the combinatorial cuckoo search algorithm.

**Pseudocode 3:**
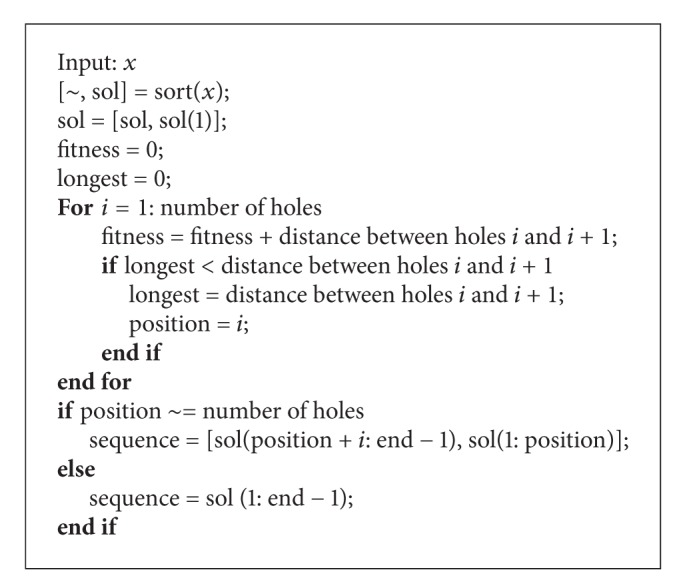
Pseudocode for fitness evaluation.

**Pseudocode 4:**
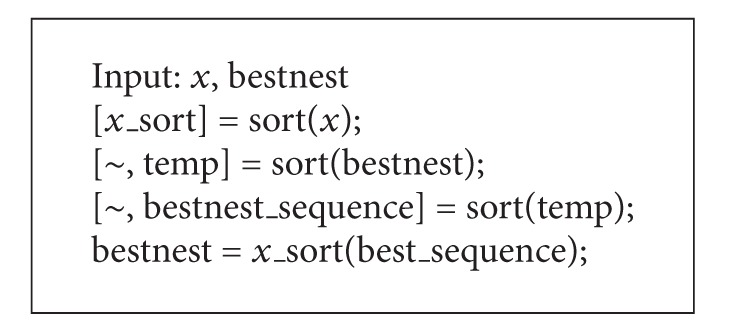
Pseudocode for inducing Lamarckian property.

The Hopfield algorithm was used for drilling path optimization [14]. The evolutionary ant colony system algorithm and artificial immune algorithm were used for the single objective and multiobjective drilling path optimization problems [15]. The greedy 2-opt algorithm was used in leather punch path optimization [5]. The genetic algorithm was used for the process route optimization [16]. The tabu search algorithm was used for holes drilling path optimization [7]. The simulated annealing algorithm was used to obtain the economical machining process [4]. The adaptive particle swarm optimization (adaptive PSO) was used by Onwubolu and Clerc [3] to solve the problem of path optimization in automated drilling operation. Walas and Askin [17] and Chauny et al. [18] proposed heuristic algorithms based on the travelling salesman problem to minimize total tool travel distance in punching operations. Using an artificial intelligence approach, Ssemakula and Rangachar [19] proposed a method to generate an operation sequence applicable to a variety of manufacturing processes.

Numerous researches have been oriented towards the development of algorithms for calculating the minimum drill path between holes [20–22]. Specifically in CNC machine, one of the earliest routing problems in holes drilling is a paper written by Sigl and Mayer [23]. They introduced the 2-opt heuristic evolutionary algorithm in solving drill routing for computer numerical control (CNC) machine. Using CNC as the subject, Qudeiri et al. [24] employed genetic algorithm (GA) in searching the optimized route for holes cutting process in CNC machine tool. Also, Ghaiebi and Solimanpur [2] have introduced an ant algorithm for holes drilling of multiple holes sizes. Medina-Rodríguez et al. [25] presented a parallel ant colony optimization algorithm to find an efficient sequence of operation for a set of holes located in a PCB board that achieves the shortest tool path. Saealal et al. [10] implemented ant colony system for PCB holes drilling route optimization problem with an objective to minimize the distance of the route chosen by the CNC machine.

Recently, cuckoo search (CS) was developed by Yang and Deb [26], which was inspired by cuckoo birds that lay eggs in the nests of other birds. Preliminary studies have shown that CS successfully outperforms the existing algorithms such as GA and PSO on various testing functions. However, there has been little development of CS in solving discrete combinatorial problems. In this paper, we will extend CS to solve combinatorial problems and suggest an approach for solving the drilling path optimization problem.

The rest of the paper is organized as follows: mathematical formulation of the route optimization in PCB holes drilling problem is presented in Section 2. The proposed cuckoo search algorithm is illustrated in Section 3.  Section 4 describes the implementation and verification results for three case study problems.  Section 5 outlines the conclusion. A comprehensive list of references ends the paper.

## 2. Routing Problem in PCB Drilling Process

When drilling a group of holes in a PCB using a CNC milling machine, the machine table is driven back and forth in the *X*-*Y* directions so that each hole is to be drilled in its designed position. The optimum drilling sequence can minimize the total table movements, thus shortening the no-cutting time and lengthening working life of the table's driving system. The *x* and *y* motions, which are realized using stepper motors, are sequential and based on the coordinates to be drilled [3]. However, Wei et al. [8] formulated the problem differently. The two perpendicular gears are allowed to move together at a certain rotational speed. Hence, the time taken for the cutting tool to move from one hole to another hole is the longest of *x* and *y*. By considering the above said machine characteristics, the problem to be solved here is to find a sequence in which the holes are to be drilled such that the tool travelling time is minimized. The travelling time is the time required for the machine to move from position *i* to position *j* and it depends strongly on the machine characteristics. In practice, usually, this travelling time cannot be computed exactly. Travelling consists of three phases: accelerating the machine, running at full speed, slowing down to a complete stop. For small distances, full speed may not be reached and we may have anomalies in the sense that a farther position can be reached faster than a nearer position. Even if a timing function is available it may be not accurate or so complicated that its evaluation takes too long for large problem instances (where we cannot store a distance matrix). Therefore, one has to be satisfied with making reasonable approximations on the true movement time. In this paper, the gears are taken to rotate at the constant speed at all times. The formula to calculate the travelling time for the drill to move from hole *i* to hole *j* is as follows.

**Case 1**

(1) $$\begin{array}{c} t_{ij}=\frac{\left|x_{i}-x_{j}\right|}{V_{x}}+\frac{\left|y_{i}-y_{j}\right|}{V_{y}}, \end{array}$$

**Case 2**

(2) $$\begin{array}{c} t_{ij}=\underset{}{\max}\left(\frac{\left|x_{i}-x_{j}\right|}{V_{x}},\frac{\left|y_{i}-y_{j}\right|}{V_{y}}\right), \end{array}$$

where *V*_*x*_ and *V*_*y*_ are the linear velocities in the *x* and *y* directions, respectively.

**Figure 1:**
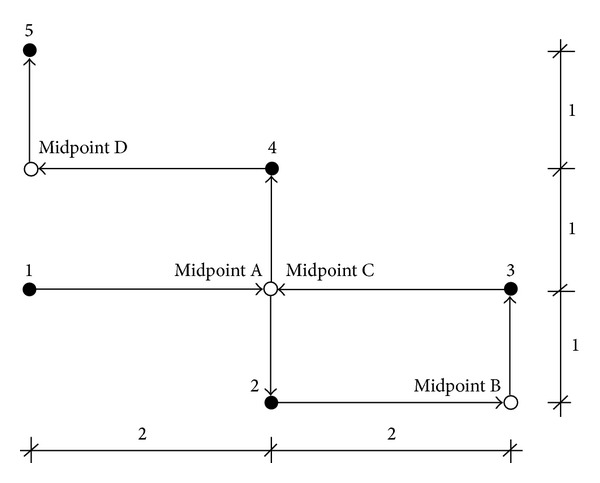
Worktable movements for Case 1.

**Figure 2:**
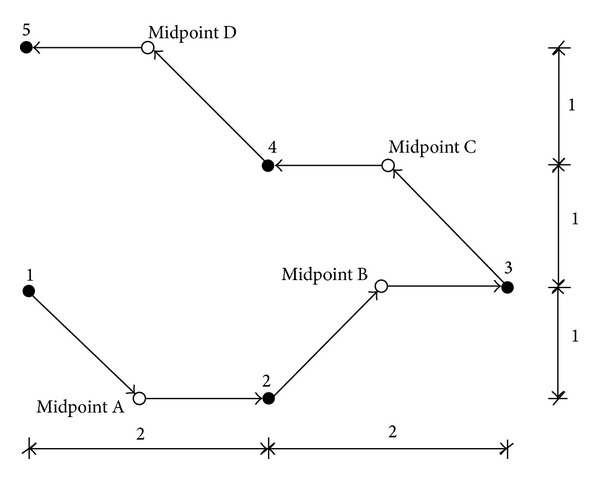
Worktable movements for Case 2.

For the first case, only one gear is allowed to turn at a time. In actuality, the time taken for the drill to travel from one hole to another hole is the same regardless of which gear is to move first. But for simplicity, the *x* gear is always chosen to move first. When the movement in the *x* direction is completed, the worktable will continue to move in the *y* direction as shown in Figure 1. For the second case, both of the gears are allowed to turn at the same time. Hence, the time taken for the travel is dependent on the gear that requires more time to turn. If the rotational speeds of the gears are the same, then the path of the drill will be as shown in Figure 2. To further simplify the problem, both *V*_*x*_ and *V*_*y*_ are taken to be the same, *V*_*x*_ = *V*_*y*_ = *V* as follows.

**Case 1**

(3) $$\begin{array}{c} t_{ij}=\frac{1}{V}\left(\left|x_{i}-x_{j}\right|+\left|y_{i}-y_{j}\right|\right). \end{array}$$

**Case 2**

(4) $$\begin{array}{c} t_{ij}=\frac{1}{V}\underset{}{\max}\left(\left|x_{i}-x_{j}\right|,\left|y_{i}-y_{j}\right|\right). \end{array}$$

Unlike the classical TSP, drilling path optimization problem does not need the cutting tool to return to its starting position. This is because drilling five holes in the sequence 1 → 2 → 3 → 4 → 5 on a workpiece has the same distance travelled and time taken as drilling five holes in the opposite sequence 5 → 4 → 3 → 2 → 1. After the best route for the cutting tool is found, say 3 → 4 → 5 → 1 → 2, the cutting tool has to just drill all five holes and stop at the last hole and reverse the sequence of drilling for the next workpiece, 2 → 1 → 5 → 4 → 3. With this in mind, the objective function of PCB drilling process can be expressed as

(5)  min⁡ ⁡∑i=1n∑j=1ntijxij,

(6)  subject  to ∏i=1nmax⁡⁡(∑j=1nxij,∑j=1nxji)=1,

where *n* is the number of holes to be drilled. Let *x*_*ij*_ be the decision variable related to the movement of the robotic arm from hole *i* to hole *j*. If there is a movement of the worktable from hole *i* to hole *j*, *x*_*ij*_ = 1; otherwise, *x*_*ij*_ = 0. The constraint equation (6) ensures that every specified hole is drilled and is only drilled once.

For solving this kind of problems, one of the simple approaches is to list all the possible paths and then compare all the path lengths to find out which one is the shortest. Unfortunately, there are too many paths. The number of possible paths increases if the number of holes in the PCB increases. For example, if 10 holes are to be drilled in a PCB, then the number of possible path is 3, 628, 800 (i.e., 10!/2) and for 20 holes, there are 2, 432, 902, 008, 176, 640, 000 (i.e., 20!/2) possible paths. Since the number of possible solutions is increasing exponentially with the number of holes to be drilled, it is time-consuming to exhaustively evaluate each and every possible solution to obtain the best solution for any problems of appreciable size. Thus, there is a need for metaheuristics to solve these problems. Metaheuristics is not guaranteed to obtain the best solution, but it is able to obtain suboptimal solution in a reasonable time [27]. In this paper, combinatorial cuckoo search algorithm is proposed to find the optimal solutions.

## 3. Combinatorial Cuckoo Search

In order to extend the cuckoo search for combinatorial discrete optimization problems, let us briefly review the interesting breed behavior of certain cuckoo species. Then, we will outline the basic ideas and steps of the proposed algorithm.

### 3.1. Cuckoo Search Behavior

The cuckoo search (CS) is one of the latest nature inspired metaheuristic algorithms developed by Yang and Deb in 2009 [26]. It was inspired by the obligate brood parasitism of some cuckoo species by laying their eggs in the nests of other host birds (of other species). Some host birds can engage in direct conflict with the intruding cuckoos. For example, if a host bird discovers that the eggs are not its own, it will either throw these alien eggs away or simply abandon its nest and build a new nest elsewhere.

### 3.2. Lévy Flights

In nature, animals search for food in a random or quasirandom manner. In general, the foraging path of an animal is effectively a random walk because the next move is based on the current location/state and the transition probability to the next location. Which direction it chooses depends implicitly on a probability which can be modeled mathematically. For example, various studies have shown that the flight behavior of many animals and insects has demonstrated the typical characteristics of Lévy flights [28].

A recent study by Reynolds and Frye [29] shows that fruit flies or *Drosophila melanogaster* explore their landscape using a series of straight flight paths punctuated by a sudden 90° turn, leading to a Lévy-flight-style intermittent scale-free search pattern. Even light can be related to Lévy flights [30]. Subsequently, such behavior has been applied to optimization and optimal search, and preliminary results show its promising capability [31].

### 3.3. Combinatorial Cuckoo Search Algorithm

We begin this section with an overview of the proposed cuckoo search algorithm. This is followed by a discussion of the original cuckoo search algorithm, including detailed descriptions of the solution encoding and decoding, evolutionary process, fitness function evaluations, and implementation.

In the original cuckoo search for continuous problems, three idealized rules are used by [26].

- Each cuckoo lays one egg at a time and dumps it in a randomly chosen nest.
- Best nests with high quality of eggs (solutions) will be carried over to the next generations.
- The number of available host nests is fixed, and a host bird can discover an alien egg with a probability *p*_*a*_ ∈ [0, 1]. In this case, the host bird can either throw the egg away or abandon the nest so as to build a completely new nest in a new location.

Based on these rules, the basic steps of the original cuckoo search can be summarized and the corresponding pseudocode is shown in Pseudocode 1.

For combinatorial problems, we modify the first and the last rule to cater for our needs.

- The best cuckoo lays many eggs at a time and dumps one egg in every nest.
- All host birds lay one egg each at a time. Each time an egg is laid, it differs from the existing egg by a fraction of *p*_*a*_ since eggs from the same host birds are similar but not identical. If the new egg has a higher quality, it will be hatched first and the host bird will discard the alien egg. If the new egg has a lower quality, the cuckoo egg will be hatched first and the young cuckoo will kick the new egg out of the nest.

In the original cuckoo search, only one egg is laid at one time. According to the life style of cuckoo birds, each cuckoo will lay more than one egg at a time in different nests. For the combinatorial cuckoo search algorithm, the cuckoo is vectorized, so that the best cuckoo can lay eggs at every nest as stated in the first rule. When laying eggs at different nests, the cuckoo will try to lay eggs that are similar to the eggs of the host nest to reduce the possibility of host birds discovering the alien egg. The second rule selects the best solutions to be passed onto the next generation and such selection ensures the algorithm converge properly. The third rule can be considered as mutation with a fraction of *p*_*a*_ where new solutions are generated according to the similarity solutions to the other solutions. These unique features work in combination, ensuring the efficiency of the proposed algorithm. Based on these three rules, the steps of the combinatorial cuckoo search can be summarized and the corresponding pseudocode is shown in Pseudocode 2.

#### 3.3.1. Encoding of Solution Representation

Cuckoo search was initially designed to solve continuous problems. In order to apply it to discrete combinatorial problems, encoding of solutions is needed. For drilling path optimization, encoded solutions are represented as a vector of numbers in the range of (−1, 1). To decode the encoded solution, the numbers are sorted in an ascending order to represent the sequence of holes to be drilled. In this paper we adopted the five principles of encoding the solutions proposed by Gen and Cheng [32]. They are explained below.

*(1) Nonredundancy.* Mapping from encoding to solutions may belong to one of the following three cases: 1-to-1 mapping, *n*-to-1 mapping, and 1-to-*n* mapping. The encoding method used in this paper is *n*-to-1 mapping. For example, an encoded solution for a five-hole problem is [0.34 −0.09 0.88 −0.66 0.91]. The tour for the drill would be the ascending order of the numbers [4 2 1 3 5]. Let us say that a random walk is performed on the encoded solution and yields [0.11 0.03 0.75 −0.39 0.85]. Although the random walk did not change the order of the holes to be visited, the random walk brought the numbers of the first two holes closer to each other, making the next iteration of random walk more probable for them to switch places.

*(2) Legality.* The permutations of an encoding should correspond to a solution. In our case, whichever way the order of the numbers in the encoded solution turns out would also yield a legal solution.

*(3) Completeness.* The encoding should allow all possible solutions in the search space to be accessible for the search operation. In our case, all solutions in the search space can be encoded.

*(4) Lamarckian Property.* Lamarckian property is simply the property of each solution being able to relate to one another regardless of the context. For example, two cuckoo eggs could have a solution of [0.44 0.21 −0.23 −0.01 0.91] and [0.08 0.21 −0.03 0.14 −0.66], respectively. The tour for these two solutions would be [3 4 2 1 5] and [5 3 1 4 2]. In the former solution, 0.21 means that the second hole is to be drilled third. Even though in the second solution 0.21 is in the same position as the first solution, 0.21 in the second solution means that the second hole is to be visited last. In this case, the solution representation is said to be no Lamarckian property. To overcome this problem, the numbers in the second egg are changed to follow the values in the first egg yet not altering the tour. In this case, the solution of the second egg will be changed to [0.21,  0.91 −0.01 0.44 −0.23] before applying any search operators. This technique induces Lamarckian property as the context will be the same.

*(5) Causality.* It is also important to have a strong causality where a small change in the encoded solutions gives a small change in the decoded solutions. This will prevent the neighborhood structure of the solutions to be destroyed with just a small change. In our case, the encoded solution exhibits strong causality as the change of an element of a vector is not destroying the neighborhood structure.

#### 3.3.2. Evaluation Matrix

The evaluation matrix is defined as an *n* × *n* matrix of the holes being drilled (where *n* is the number of holes to be drilled) for which we seek the optimal drill path to reduce the travelling time for table movements. The distances between every two points are computed to generate the evaluation matrix.

**Figure 3:**
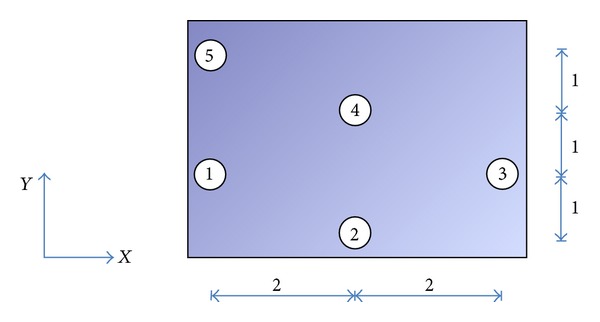
Description of the points to be drilled.

For a simple drilling path optimization problem of 5 holes in a component as shown in Figure 3, to be drilled using a CNC drilling machine such that the coordinates for *x* = [1,3, 5,3, 1] and *y* = [2,1, 2,3, 4]. The corresponding evaluation matrix for worktable movements (Case 1 and Case 2) are shown in the following matrices, respectively.

Evaluation matrix for Case 1 is as follows:

(7) [034      303      430      323      25632      25      36      03      30].

Evaluation matrix for Case 2 is as follows:

(8) [024      202      420      222      23422      23      24      03      20].

#### 3.3.3. Initial Population

The initial population of the eggs is randomly generated with a range of (−1, 1). Of course, any randomization can be applied here. But we use random numbers drawn from the standard normal distribution and limit to (−1,1) by taking only the sign and the decimal part of the numbers.

#### 3.3.4. Fitness Evaluation

As mentioned earlier, the encoded solution will be sorted in an ascending order to represent the sequence of holes to be drilled. However, for evaluating the fitness, the first hole will be repeated again at the end of the sequence. The distances between each of the holes are calculated and added together, and the longest distance among all the distances will be subtracted to represent the distance travelled. This is done because the drill bit does not need to travel back to its starting position. The corresponding hole to the subtracted distance becomes the starting position. The pseudocode for fitness evaluation is given in Pseudocode 3.

#### 3.3.5. Best Cuckoo Lays Eggs in All Nests

Lévy flight is performed to generate new solution stochastically:

(9) $$\begin{array}{c} x_{i}\left(t+1\right)=x_{i}\left(t\right)+\alpha ⊕\text{Lévy}\left(\beta \right)\text{,} \end{array}$$

where *α* > 0 is the step size which should be related to the scale of the problem of interest. In order to accommodate the difference between solution qualities, in this paper we adopt the same value used by Yang and Deb [33]:

(10) $$\begin{array}{c} \alpha =\alpha_{0}\left[x_{j}\left(t\right)-x_{i}\left(t\right)\right]\text{,} \end{array}$$

where *α*_0_ is a constant, while the term in the bracket corresponds to the difference of two selected solutions. This mimics the fact that similar eggs are less likely to be discovered and newly generated solutions are proportional to their differences. The product ⊕ means entrywise multiplication. Lévy flight is essentially a Markov chain in which the random steps are drawn from the Lévy distribution [27].

The generation of the Lévy distribution can be achieved by Mantegna's algorithm. Mantegna's algorithm produces random noise according to a symmetric Lévy stable distribution [33]. A symmetric Lévy stable distribution is ideal for Lévy flights as the direction of the flight should be random [27].

In Mantegna's algorithm, the step length can be calculated by

(11) $$\begin{array}{c} \text{Lévy}\left(\beta \right)\sim \frac{u}{{\left|v\right|}^{1/\beta }}\text{,} \end{array}$$

where *u* and *v* are drawn from normal distributions. That is,

(12) $$\begin{array}{c} u\sim N\left(0,\sigma_{u}^{2}\right),\;\;v\sim N\left(0,\sigma_{v}^{2}\right)\text{,}\\ \sigma_{u}={\left\{\frac{\Gamma \left(1+\beta \right)\sin\left(\pi \beta /2\right)}{\Gamma \left[\left(1+\beta \right)/2\right]\beta 2^{\left(\beta -1\right)/2}}\right\}}^{1/\beta },\;\sigma_{v}=1, \end{array}$$

where the distribution parameter *β* ∈ [0.3,1.99] [34].

For the best cuckoo laying eggs, the step size, *α*, used is

(13) $$\begin{array}{c} \alpha =\alpha_{c}\left[\text{bestnest}\left(t\right)-x_{i}\left(t\right)\right], \end{array}$$

where *α*_*c*_ is a constant.

To induce the Lamarckian property, the value of the best nest is altered to match the values in each nest. The implementation of this is in Pseudocode 4.

#### 3.3.6. Host Birds Lay Eggs

New eggs laid by host birds are generated using the Lévy flights:

(14) $$\begin{array}{c} x_{i}\left(t+1\right)=x_{i}\left(t\right)+K⊕\alpha ⊕\text{Lévy}\left(\beta \right)\text{,} \end{array}$$

where *K* is a vector of 0 and 1. The value of each *K* is obtained with

(15) $$\begin{array}{c} K=\{\begin{array}{cc} 1 & \text{if rand}<p_{a}\\ 0 & \text{else,} \end{array} \end{array}$$

where rand is the random number obtained from a uniform distribution in the range of [0,1]. The step size, *α*, used is

(16) $$\begin{array}{c} \alpha =\alpha_{h}\left[x_{i}\left(t\right)-x_{j}\left(t\right)\right]\text{,} \end{array}$$

where *α*_*h*_ is a constant, and both *x*_*i*_ and *x*_*j*_ are randomly selected nests from the population. The same method that is in Pseudocode 4 is used to induce Lamarckian property for both of the randomly chosen nests.

## 4. Implementation and Verification

The proposed CS algorithm is implemented in MATLAB R2011b on Intel Core 2 CPU 6600@2.40 GHz with 2.00 GB RAM and 32-bit processor. The performance of the algorithm is verified by solving three case study problems taken from the literature, namely, workpieces 1 and 2 [9] and workpiece 3 [8]. The algorithm parameters, *p*_*a*_, *α*_*c*_, and *α*_*h*_, are varied with a range of [0,1], [0,2], and [0,2], respectively, with *G* = 4000 and *n* = 50, and fine-tuned using the original CS. From test phase simulations, we found that the optimal values for the parameters are *p*_*a*_ = 2/(number of holes − 2) and *α*_*c*_ = 3/number of holes, respectively. Generally, *α*_*h*_ is best to be in the range of [1,2] but does not affect the quality of the solutions drastically.

In solving the case study problems, (3) and (4) are used as the evaluation function of the algorithm when *V* = 1 mm s^−1^; parameters were fixed as *p*_*a*_ = 2/(number of holes − 2), *α*_*c*_ = 3/number of holes, and *α*_*h*_ = 1; set the generation number to 10 000 for workpiece 1 and workpiece 2 and 20 000 for workpiece 3; set the population number to 50 for workpiece 1 and workpiece 2 and 100 for workpiece 3. The algorithm was run for 1000 times and the results were taken. The data verification results of workpiece 1 (10 holes problem) and workpiece 2 (15 holes problem) used by Zhu and Zhang [9] and workpiece 3 (50 holes problem) taken from [8] for both cases are shown in Tables 1, 2, and 3, respectively.

From Tables 1, 2, and 3, it is worthwhile to mention that there is an enormous saving in time of 87.3 seconds, 60.0 seconds, and 370.0 seconds in workpieces 1–3, respectively. Previous researchers mostly followed Case 1 in drilling path optimization. The optimal sequence obtained by CS for the three workpieces in both cases is shown in Table 4. The results obtained using CS are compared with PSO [9] and ACS [10] for workpiece 1 and workpiece 2 and presented in Tables 5 and 6.

For the CS algorithm, population number of 50 searches 100 solutions in one iteration. Hence, for a more meaningful comparison, the search ratio is used rather than the number of iterations.

The search ratio equation is described as

(17) $$\begin{array}{c} \frac{\text{Total searched solutions}}{\text{Solution space}}=\text{Search ratio}. \end{array}$$

**Figure 4:**
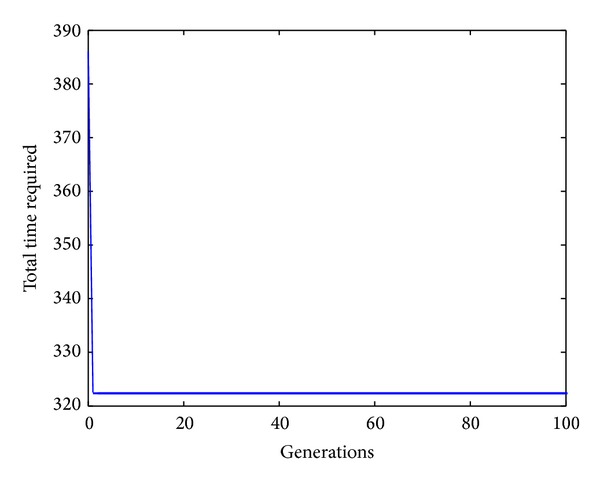
The convergence curve for workpiece 1.

**Figure 5:**
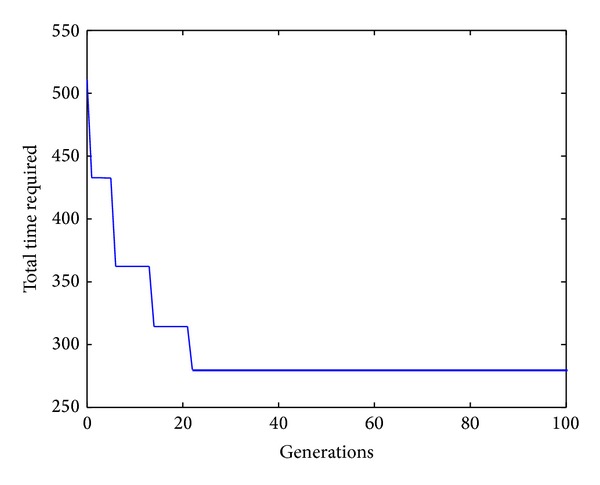
The convergence curve for workpiece 2.

**Figure 6:**
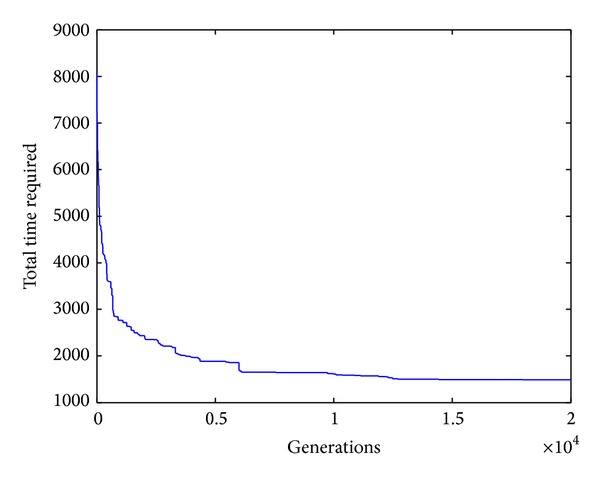
The convergence curve for workpiece 3.

According to (17), the least search ratio is simply the ratio between the least iteration number during global convergence and the solution space, while the average search ratio is the ratio between the average iteration number during global convergence and the solution space. It is well known from Tables 5 and 6 that the CS has an average search ratio of 0.248% and 0.000 001 32% for workpiece 1 and workpiece 2, respectively. For workpiece 1, the CS performs well in all aspects. For workpiece 2, CS has the least average iteration during global convergence. The rate of convergence of CS algorithm for workpieces 1–3 can be seen in Figures 4, 5, and 6, respectively.

## 5. Conclusion

In this paper, combinatorial cuckoo search algorithm was proposed and implemented for PCB holes drilling route optimization problems with objective to minimize the time taken of the route chosen by the CNC machine. The proposed CS is quite straightforward and easy to implement. A number of experiments were carried out to fine-tune the CS algorithm parameters. The performance of the proposed algorithm is tested and verified with three case studies from the literature. The computational experience conducted in this research indicates that the proposed algorithm is capable of efficiently finding the optimal path for PCB holes drilling process. The results obtained indicate that the CS algorithm has the characteristics of easy realization, fast convergence speed, low search ratio, and better global converging capability. Despite already obtaining promising results, further works might include hybridizing with other algorithms like genetic algorithm to improve global exploration to handle larger size problems.

## Figures and Tables

**Table 1:** Verification results for workpiece 1.

Parameters	Worktable movements	
	Case 1	Case 2
The least iteration number during global convergence	1	1
The average iteration number during global convergence	18	13
The most iteration number during global convergence	57	40
The optimal time required	322.5	235.2

**Table 2:** Verification results for workpiece 2.

Parameters	Worktable movements	
	Case 1	Case 2
The least iteration number during global convergence	23	30
The average iteration number during global convergence	429	375
The most iteration number during global convergence	2349	2092
The optimal time required	280.0	220.0

**Table 3:** Verification results for workpiece 3.

Parameters	Worktable movements	
	Case 1	Case 2
The least time required	1489.742	1119.706
The average time required	1805.262	1338.650
The most time required	2037.243	1487.126
Percentage error for the least time required	2.02%	1.06%
Percentage error for average time required	23.63%	20.82%
Percentage error for most time required	39.52%	34.23%
The optimal time required	1460.216	1107.927

**Table 4:** Optimal sequence obtained by CS for the three workpieces.

Case study problem	Optimal sequence
Workpiece 1 (10-hole problem)	
Case 1	3 2 1 6 7 8 5 4 9
Case 2	3 2 1 6 7 8 5 4 9

Workpiece 2 (15-hole problem)	
Case 1	10 11 12 9 6 5 1 2 3 4 7 8 13 14
Case 2	1 5 6 4 2 3 7 9 11 10 12 8 13 14

Workpiece 3 (50-hole problem)	
Case 1	2 39 40 41 37 38 32 31 34 33 35 36 30 29 27 28 25 26 48 47 45 44 43 46 24 21 22 23 19 20 14 15 16 17 18 13 8 7 10 9 11 12 2 1 4 3 5 6 49
Case 2	6 45 44 43 48 47 26 25 27 28 29 30 31 32 33 34 35 36 37 38 39 40 41 42 1 2 3 4 5 6 49 7 8 9 10 11 12 13 14 15 16 17 18 19 20 21 22 23 24

**Table 5:** Performance comparison of CS with variants of PSO on workpiece 1.

	Basic PSO	GC PSO			CS
	*ω* = 1.0	*ω* = 0.0	*ω* = 0.5	*ω* = 1.0	—
Population number	100	100	100	100	50
The least iteration number during global convergence	9	1	5	4	1
The average iteration number during global convergence	20	1251	646	1620	18
The least number of solutions searched	900	100	500	400	100
The average number of solutions searched	2000	125100	64600	162000	1800
The least search ratio	4.96*E* − 1%	5.51*E* − 2%	2.76*E* − 1%	2.20*E* − 1%	5.51*E* − 2%
The average search ratio	1.10%	68.9%	35.6%	89.3%	9.92*E* − 1%
Length of optimal path (mm)	322.5	322.5	322.5	322.5	322.5

**Table 6:** Performance comparison of CS with variants of PSO and ACS on workpiece 2.

	Basic PSO	GC PSO			ACS	CS
	*ω* = 1.0	*ω* = 0.0	*ω* = 0.5	*ω* = 1.0	—	—
Population number	100	100	100	100	25	50
The least iteration number during global convergence	93	815	10	110	193	23
The average iteration number during global convergence	847	3806	1620	1764	1037	429
The least number of solutions searched	9300	81500	1000	11000	4825	2300
The average number of solutions searched	84700	380600	162000	176400	25925	42900
The least search ratio	2.13*E* − 5%	1.87*E* − 4%	2.29*E* − 6%	2.52*E* − 5%	1.11*E* − 5%	5.28*E* − 6%
The average search ratio	1.94*E* − 4%	8.73*E* − 4%	3.72*E* − 4%	4.05*E* − 4%	5.95*E* − 5%	9.84*E* − 5%
Length of optimal path (mm)	280.0	280.0	280.0	280.0	280.0	280.0
